# Mechanics of tensegrity cell units incorporating asymmetry and insights into *mollitaxis*

**DOI:** 10.1098/rsif.2023.0082

**Published:** 2023-05-17

**Authors:** E. Benvenuti, G. A. Reho, S. Palumbo, M. Fraldi

**Affiliations:** ^1^ Engineering Department, University of Ferrara, Ferrara, Italy; ^2^ Department of Structures for Engineering and Architecture, University of Napoli Federico II, Napoli, Italy

**Keywords:** cell mechanobiology, durotaxis, cellular tensegrity, nonlinear elasticity

## Abstract

The mechanical response of a contractile cell anchored to the substrate through focal adhesions is studied by means of an asymmetric pre-strained tensegrity structure obeying a neo-Hookean stress–strain law. The aim is to assess the influence of overall asymmetric contraction on the cell durotaxis and on the growth of the focal adhesion plaque. The asymmetric kinematics of the system is obtained in two ways, that is by assuming a gradient of the substrate stiffness and through asymmetric buckling. Equivalent springs are purposely considered to represent the stiffness of the ensemble formed by the substrate, the focal adhesion plaque and the integrin ligands. Then, contraction results from elastic strains induced by competing polymerization and actomyosin contraction. The cell mechanical response in terms of durotaxis and its coupling with focal adhesion plaque growth is finally analysed with respect to the effects of asymmetry, gaining some insights into how this asymmetry could participate to redirect cell migration, both in terms of durotaxis and mollitaxis.

## 1. Introduction

The mechanical response of a pre-strained tensegrity structure is proposed to capture the contractile behaviour of a cell anchored to the substrate through focal adhesions. Particularly, the interplay among cell contractility, asymmetric buckling and substrate stiffness gradients and their influence on cell mobility is investigated. The forces exerted by the cell on the substrate are shown to induce durotaxis and influence the growth of the focal adhesion plaque-like structure linking the cell to the extracellular matrix.

The internal organization and the overall shape of the cell are controlled by both the cytoskeleton system and the extracellular matrix. Their interaction stimulates fibronectin fibril assembly and fibres orientation [1,2], which, in turn, control the cell shape through the modulation of the cytoskeletal stiffness and influence the strength of the integrin–cytoskeleton linkages [3,4]. These linkages form the focal adhesion complexes through a process regulated by trans-membrane receptors of the integrin family [5] and different types of adapter proteins, e.g. talin, vinculin and paxillin, which constitute the adhesion plaque.

The growth and development of the focal adhesions is affected by the substrate stiffness. Focal adhesion complexes that grow on a stiffer substrate are more stable and elongated than those grown on soft substrates [6,7]. Cell locomotion is key to morphogenesis [8], immunological defence, wound healing [9] and tumour metastasis [10,11]. Locomotion is made possible owing to the formation and release of the cell–substrate anchorages exerted by the focal adhesions, through a sequence of stages where morphological polarization of the cell takes place by the frontal protrusion of the lamellipodium, cell contractility and subsequent release of the adhesions at the rear of the cell [12].

Both cytoskeleton and substrate stiffness play a relevant role in cell locomotion [13,14]. When driven by the substrate stiffness gradients, cell locomotion is often referred to as durotaxis.

Durotaxis indicates the attitude of cells to migrate by probing substrate rigidity gradients, and it is a widely accepted mechanobiological mechanism. First observed *in vitro* by Lo *et al.* [15], durotaxis has been very recently detected *in vivo* by Shellard & Mayor [16], who showed how gradients of chemical and mechanical signals cooperate to achieve efficient directional cell migration. Durotaxis manifests as a consequence of the fact that the cell contractility generates forces on the substrate, and these forces must be balanced either at the cell periphery or throughout the cell–substrate interface [17,18]. While, in many cases, cells steer toward stiffer substrates [19,20], some may migrate towards softer substrates [21]. Hence the term negative durotaxis, or mollitaxis, has been coined to indicate the tendency to move toward softer environments. A spontaneous switch from positive to negative durotaxis has been observed in neurons. It can also be induced in other cells by talin- and vinculin-mediated focal adhesions formation disruption [21] because the force exerted by the cell attains its maximum in a certain substrate stiffness range intermediate between soft and hard substrates.

Cell locomotion is inherently directional, polarized and symmetry-breaking [22]. Asymmetry also results from curvotaxis [23], i.e. when cells respond to curvature variations, or from pre-existing external and/or internal defects and stimuli [24]. Furthermore, asymmetric reorganization of cytoskeletal architecture can be triggered by fluctuations of density and local concentration of signalling molecules and focal adhesions bonds, and by configurational heterogeneities induced by polymers alignment and orientation. Other symmetry-breaking factors result from the space-variability of the mechanical response of the cytoskeletal network to external or internal forces. As for the role played by the asymmetry in determining the behaviour of the main cytoskeleton components, actin filaments and microtubules, and their networks, which are crucial in the generation and transmission of pushing or pulling forces on the substrate, are structurally and kinetically asymmetric, as they are polarized, and polarization implies that one end grows faster than the other one so that structural symmetry is broken [25,26].

The present contribution focuses on the development of a mechanical tensegrity model of an adherent cell exhibiting overall asymmetric contraction, introduced by considering stiffness gradients of the underlying substrate and asymmetric buckling of the compressed elements. The aim is to assess with a simple physical model whether and to which extent asymmetry and stiffness gradients influence cell mobilization and focal adhesions’ assembly and disassembly. The cytoskeleton is purposely reduced to its main components, that is actin filaments and microtubules [27] forming a contractile mechanical system obeying the so-called tensegrity self-equilibrium principle whose prominent role in cell mechanics was first highlighted in the seminal papers by Ingber *et al.* [3,28,29]. A comprehensive review of the key aspects of tensegrity structures in cellular biophysics is available in [30]. Recent authors’ studies have assessed the key aspects inherent to buckling phenomena and nonlinear elasticity in tensegrities for living cells [31,32]. In the present contribution, the system contraction is triggered by means of inelastic pre-strains, that simulate pre-contraction and pre-polymerization, analogously to the actomyosin model previously developed by the authors in [33,34]. The cell contractile activity produces two equal forces at the leading and trailing edges of the cell where the plaques of the focal adhesions are considered to be located. These forces, in turn, induce a thermodynamically consistent polymerization/depolymerization process of the focal adhesion plaques. Based on previous works by Benvenuti *et al.* [33] and Palumbo *et al.* [34], whose main aim was assessing the role of cytoskeleton pre-contraction and pre-polymerization, possibly inducing buckling, in the development of adhesion sites in stationary (i.e. non-migrating) cells, the present contribution then addresses durotaxis. It is worth noting that, as shown in the following, both ‘classical’ positive durotaxis and more recently unveiled examples of mollitaxis can be retraced by means of the proposed essential model, the switching from one mechanism to the other depending on the combination of geometrical asymmetry, stiffness gradients and inelastic pre-strains. Advantageously, the present model allows us to parametrically investigate the effect of a wide range of asymmetric configurations and stiffness gradients on the cell kinematics and how these affect the process of assembly and disassembly of the focal adhesion plaques subjected to the force exerted by the system.

The remainder of the paper opens with the fundamentals of the proposed asymmetric tensegrity model in terms of components and relevant mechanical behaviour (§2). Section 3 briefly discusses the choice of the pre-strains and the parameters adopted in the numerical results. Results are then shown in §4 focusing on the role played by asymmetry on the occurrence of positive and negative durotaxis for certain substrate stiffness gradients. In §5, critical asymmetry ratios that mark the transition from positive to negative durotaxis are reported for variable pre-strains. The way the system induces forces and growth fluxes on the plaque based on asymmetry and stiffness gradients is assessed in §6. Finally, the role of plaque geometry and average substrate stiffness is discussed in §7.

## 2. An asymmetric tensegrity model for incipient cell locomotion

The present section focuses on the equilibrium problem of a geometrically essential, nonlinear, buckling tensegrity model as a means to assess the influence of asymmetric kinematics and stiffness gradients on cell durotaxis and focal adhesion plaque growth. The main features of the tensegrity model are: a set of equivalent springs at both sides of the tensegrity representing the stiffness of the ensemble formed by the substrate, the focal adhesion plaque and the integrin ligands; polymerization and contraction pre-strains applied to the system, which reacts transmitting a mechanical force to the edge springs; asymmetric buckling obtained by asymmetric geometry and considering substrate stiffness gradients.

### 2.1. Other durotactic mechanical models

Durotaxis is the attitude of cells to move driven by the substrate stiffness gradients. Cell contractility is intrinsic to durotaxis [17].

Physical models of durotaxis include [16,35] the model of the persistent walk assuming that cells move more persistently on stiffer substrates than on softer ones [36]. Other thermodynamically consistent models reproduce stronger attachments on stiffer substrates, resulting in a net forward cell movement [22].

Based on the so-called motor–clutch hypothesis [37], motor–clutch models allow for myosin II fibres to transmit equal forces to the stiff and soft parts of the graded substrate through integrin complexes. The substrate reacts with larger displacements at the trailing edge, thereby pushing the cell centre towards the front [20,35,38]. Motor–clutch model results consistent with both positive and negative durotaxis have been obtained [18,21].

### 2.2. Mechanical system

**Figure 1.**
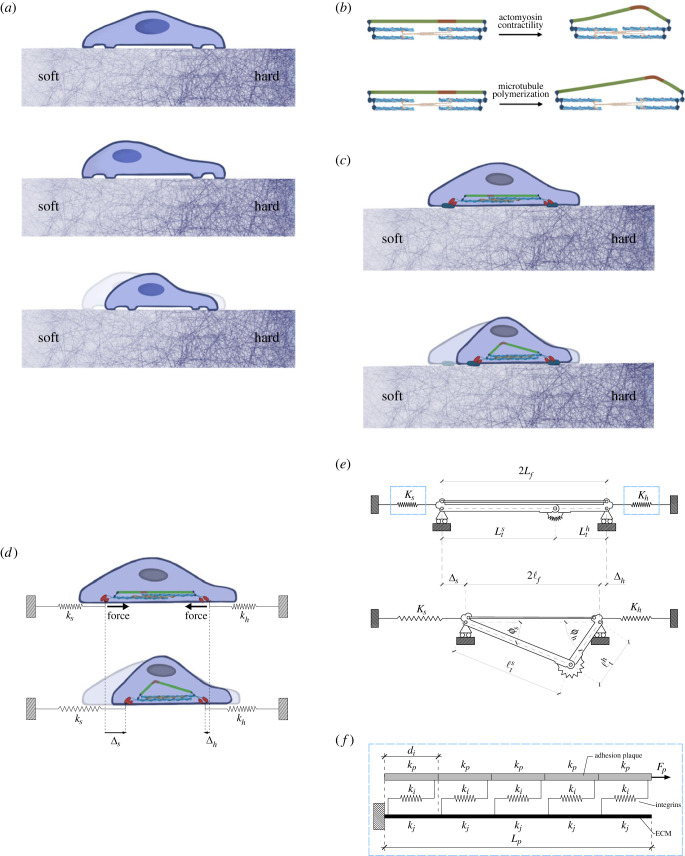
(*a*) Sketch of a contractile adherent cell on a substrate with a stiffness gradient. (*b*) Representation of the cytoskeletal system behaviour subjected to actomyosin contraction or microtubule polymerization. (*c*) Sketch of an adherent cell on a substrate with a stiffness gradient subjected to pre-contraction. (*d*) Representation of an adherent cell with an embedded contractile system bonded to equivalent springs representing the soft and hard substrates. (*e*) Asymmetric mechanical model of the adherent cell in its stress-free reference state and in its deformed configuration. (*f*) Structural scheme adopted for the focal adhesion–extracellular matrix complex with *j* = *h*, *s*.

To clarify the adopted mechanical framework, figure 1*a* represents the contraction of an adherent cell during classical positive durotaxis on a substrate whose stiffness varies from soft to hard. Here, locomotion is modelled by means of a mechanical system made by a buckling-prone element, representative of the compressed microtubular components of the cell, and a stress-fibre-like element subjected to tensile states, these two elements providing the simplest tensegrity paradigm [31]. In this model, further extending the approach developed in recent authors’ contributions [33,34], cell kinematics is assumed to be triggered by actomyosin contraction of the stress fibres combined with polymerization of the buckling-prone component as shown in figure 1*b,c*. Also, as cells’ locomotion is a polarized process, it is inherently asymmetric. This asymmetry is here associated with asymmetric buckling of the compressed component.

The cytoskeleton is simulated through the tensegrity system shown in figure 1*e*. In the tensegrity, an element representative of the actomyosin complex is taken in parallel with another element corresponding to the microtubule [33,34]. The former can only elastically elongate or inelastically contract without bending, while the latter is a compression-bearing buckling-prone element that can also polymerize. In this way, such a model accounts for the main features of the mechanical behaviour of a eukaryotic cell, including actomyosin contraction and polymerization. Furthermore, as mentioned above, asymmetric buckling of the microtubule is assumed. This is modelled as induced by a defect of the microtubule, which consists in shifting the elastic hinge allowing for buckling with respect to the middle point. Therefore, by considering the fibre length equal to 2*L*_*f*_, since we assume that the hinge position varies along the horizontal axis, it will divide the microtubule into two subunits of, generally, different lengths, $L_{t}^{h}$, and $L_{t}^{s}$, these being the length of the microtubular part close to the hard-substrate and the soft-substrate sides, respectively. To assess the role of symmetry breaking on the microtubule buckling, the asymmetry ratio *η* ∈ ]0, +∞[ is introduced as the ratio $\eta =L_{t}^{s}/L_{t}^{h}$. Finally, the springs located at the two edges of the tensegrity represent the stiffness of the focal adhesion–extracellular matrix complexes. To introduce the substrate stiffness gradients, the springs possess different stiffnesses identified by *K*_*h*_ and *K*_*s*_ for the hard and soft portion of the substrate, respectively. The expressions for these stiffnesses are derived from Cao *et al.* [39] on the basis of figure 1*f*, as also previously done in [33,34,40]. Note that, therein, the stiffness of the substrate is given by *k*_*h*_ or *k*_*s*_ in the case of hard and soft substrates, respectively.

As an effect of the mechanosensitivity of the devised structural system, the displacement $\Delta_{s}$ at the edge lying upon the softer substrate will be generally different from $\Delta_{h}$ detected at the edge placed on the hard part of the substrate. The cell net displacement $\Delta_{N}$ can be hence computed as [35]

2.1 $$\Delta_{N}=\Delta_{s}-\Delta_{h}.$$

The contractile system illustrated in figure 1*d* exemplifies the positive durotaxis concept with the cell advancing towards the stiffer side [18]. It will be demonstrated in the subsequent developments that the present model accomplishes both positive and negative durotaxis as a consequence of the balance between contraction and polymerization pre-strains. As a possible extension, other pre-tensioning strategies could be used, such as the effective cable-actuation process for three-dimensional tensegrities proposed in [41].

### 2.3. Formulation of the equilibrium problem

The microtubule and the fibre bundles are considered to be inelastically pre-stretched through polymerization $\lambda_{t}^{*}\in [1,+\infty [$ and pre-contraction $\lambda_{\;f}^{*}\in \;]0,1]$, respectively. These pre-stretches stimulate the system to respond with elastic incremental stretches ${\hat{\lambda }}_{\;f}\in [1,+\infty [$, ${\hat{\lambda }}_{t}^{h}\in \;]0,1]$ and ${\hat{\lambda }}_{t}^{s}\in \;]0,1]$. Consequently, the total stretch *λ*_*f*_ of the stress fibres bundle turns out to be given via multiplicative superposition of the inelastic contractile stretch $\lambda_{\;f}^{*}$ and the elastic one ${\hat{\lambda }}_{\;f}$:

2.2 $$\lambda_{\;f}={\hat{\lambda }}_{\;f}\lambda_{\;f}^{*}.$$

By analogy, the total stretches $\lambda_{t}^{h}$ and $\lambda_{t}^{s}$ of the microtubule subunits ‘*h*’ and ‘*s*’ are obtained via multiplicative superposition of the inelastic stretch $\lambda_{t}^{*}$ and the elastic ones, ${\hat{\lambda }}_{t}^{h},{\hat{\lambda }}_{t}^{s}$:

2.3 $$\lambda_{t}^{h}={\hat{\lambda }}_{t}^{h}\lambda_{t}^{*},\;\lambda_{t}^{s}={\hat{\lambda }}_{t}^{s}\lambda_{t}^{*}.$$

In the initial configuration shown at the top of figure 1*e*, the length of the fibre is

2.4 $$2L_{\;f}=L_{t}^{h}+L_{t}^{s}=L_{t}^{h}(1+\eta ),$$

where $L_{t}^{s}\;:=\eta L_{t}^{h}$ and *η* is the asymmetry ratio, so that $L_{\;f}=L_{t}^{h}(1+\eta )/2$. As a consequence of equations (2.2) and (2.3), the current semi-length of the fibre and the length of the microtubule subunits can be expressed as

2.5 $$\ell_{\;f}=L_{\;f}\lambda_{\;f}^{*}\;{\hat{\lambda }}_{\;f},\;\ell_{t}^{h}=L_{t}^{h}\lambda_{t}^{*}\;{\hat{\lambda }}_{t}^{h},\;\ell_{t}^{s}=\eta L_{t}^{h}\lambda_{t}^{*}\;{\hat{\lambda }}_{t}^{s},$$

the latter lengths being related by the sine rule as follows:

2.6 $$\ell_{t}^{s}\sin\phi_{s}=\ell_{t}^{h}\sin\phi_{h}.$$

The replacement of equation (2.5) in equation (2.6) and the use of basic trigonometric identities allow writing

2.7 $$\cos\phi_{s}=\frac{\;{\hat{\lambda }}_{t}^{h}}{\eta \;{\hat{\lambda }}_{t}^{s}}\;f_{1}(\phi_{h},\;{\hat{\lambda }}_{t}^{h},\;{\hat{\lambda }}_{t}^{s}),$$

where

2.8 $$\;f_{1}(\phi_{h},\;{\hat{\lambda }}_{t}^{h},\;{\hat{\lambda }}_{t}^{s})\;:={\left[\frac{\eta^{2}{\left(\;{\hat{\lambda }}_{t}^{s}\right)}^{2}}{{\left(\;{\hat{\lambda }}_{t}^{h}\right)}^{2}}-1+\cos^{2}\phi_{h}\right]}^{1/2}.$$

By geometric consistency, the final length of the fibre

2.9 $$2\ell_{\;f}=\ell_{t}^{h}\cos\phi_{h}+\ell_{t}^{s}\cos\phi_{s}$$

is first rewritten in terms of the stretches as

2.10 $$2L_{\;f}\lambda_{\;f}^{*}\;{\hat{\lambda }}_{\;f}=L_{t}^{h}\lambda_{t}^{*}\;{\hat{\lambda }}_{t}^{h}\cos\phi_{h}+\eta L_{t}^{h}\lambda_{t}^{*}\;{\hat{\lambda }}_{t}^{s}\cos\phi_{s},$$

and, subsequently, cast as a function of the asymmetry parameter *η* as follows:

2.11 $$L_{t}^{h}(1+\eta )\lambda_{\;f}^{*}\;{\hat{\lambda }}_{\;f}=L_{t}^{h}\lambda_{t}^{*}\;{\hat{\lambda }}_{t}^{h}\cos\phi_{h}+\eta L_{t}^{h}\lambda_{t}^{*}\;{\hat{\lambda }}_{t}^{s}\left(\frac{\;{\hat{\lambda }}_{t}^{h}}{\eta \;{\hat{\lambda }}_{t}^{s}}\;f_{1}(\phi_{h},\;{\hat{\lambda }}_{t}^{h},\;{\hat{\lambda }}_{t}^{s})\right).$$

Therefore, the incremental fibre stretch reads

2.12 $$\begin{array}{rl} \;{\hat{\lambda }}_{\;f} & =\frac{\lambda_{t}^{*}\;{\hat{\lambda }}_{t}^{h}}{\lambda_{\;f}^{*}(1+\eta )}(\cos\phi_{h}+\;f_{1}(\phi_{h},\;{\hat{\lambda }}_{t}^{h},\;{\hat{\lambda }}_{t}^{s}))\\ & =\frac{\lambda_{t}^{*}\;{\hat{\lambda }}_{t}^{h}}{\lambda_{\;f}^{*}(1+\eta )}\;f_{2}(\phi_{h},\;{\hat{\lambda }}_{t}^{h},\;{\hat{\lambda }}_{t}^{s}), \end{array}$$

where the position

2.13 $$\;f_{2}(\phi_{h},\;{\hat{\lambda }}_{t}^{h},\;{\hat{\lambda }}_{t}^{s})\;:=\;f_{1}(\phi_{h},\;{\hat{\lambda }}_{t}^{h},\;{\hat{\lambda }}_{t}^{s})+\cos\phi_{h}$$

has been set. From here on, the dependence of functions $\;f_{1}(\phi_{h},\;{\hat{\lambda }}_{t}^{h},\;{\hat{\lambda }}_{t}^{s})$ and $\;f_{2}(\phi_{h},\;{\hat{\lambda }}_{t}^{h},\;{\hat{\lambda }}_{t}^{s})$ on the Lagrangian parameters is dropped for conciseness. Compatibility also prescribes that

2.14 $$\;\Delta =\Delta_{s}+\Delta_{h}=2(L_{\;f}-\ell_{\;f})$$

2.15 $$=2L_{\;f}(1-\lambda_{\;f}^{*}\;{\hat{\lambda }}_{\;f})=L_{t}^{h}(1+\eta -\lambda_{t}^{*}\;{\hat{\lambda }}_{t}^{h}\;f_{2}),$$

where

2.16 $$\Delta_{s}=\alpha \Delta ,\;\Delta_{h}=(1-\alpha )\Delta ,\;\alpha \in [0,\;1]$$

are the displacements of the focal adhesions–ECM complex at the soft-substrate and hard-substrate sides, respectively.

By assuming that both microtubule and fibres obey an incompressible neo-Hookean law [32], the hyperelastic energies $U_{t}^{h}$, $U_{t}^{s}$ and $U_{\;f}$ stored by each of the subunits of the microtubule and by the fibre, respectively, are consequently prescribed as

2.17a $$U_{t}^{h}=\frac{K_{t}L_{t}^{h}\lambda_{t}^{*}}{6}\left[{\left(\;{\hat{\lambda }}_{t}^{h}\right)}^{2}+\frac{2}{\;{\hat{\lambda }}_{t}^{h}}-3\right],$$

2.17b $$U_{t}^{s}=\frac{K_{t}L_{t}^{s}\lambda_{t}^{*}}{6}\left[{\left(\;{\hat{\lambda }}_{t}^{s}\right)}^{2}+\frac{2}{\;{\hat{\lambda }}_{t}^{s}}-3\right]\;\;$$

2.17c $$\mathrm{and}\;\;\;U_{\;f}=\frac{K_{\;f}L_{\;f}\lambda_{\;f}^{*}}{6}\left[{\left(\;{\hat{\lambda }}_{\;f}\right)}^{2}+\frac{2}{\;{\hat{\lambda }}_{\;f}}-3\right],$$

where *K*_*t*_ = *E*_*t*_ *A*_*t*_ and *K*_*f*_ = *E*_*f*_ *A*_*f*_, *E*_*t*_ and *E*_*f*_ being the Young modulus for the microtubule and stress fibre, respectively, while *A*_*t*_ and *A*_*f*_ being the corresponding cross-sectional areas.

For the sake of the solution to the problem at hand, the fibre energy is rewritten in terms of the elastic microtubule stretches as follows:

2.18 $$\begin{array}{rl} U_{\;f} & =\frac{K_{\;f}L_{t}^{h}\lambda_{\;f}^{*}(1+\eta )}{12}\{{\left[\frac{\lambda_{t}^{*}\;{\hat{\lambda }}_{t}^{h}}{\lambda_{\;f}^{*}(1+\eta )}\;f_{2}\right]}^{2}\\ & \;+\frac{2\lambda_{\;f}^{*}(1+\eta )}{\lambda_{t}^{*}\;{\hat{\lambda }}_{t}^{h}}{\;f_{2}}^{-1}-3\}. \end{array}$$

Moreover, the energies $U_{s}$ and $U_{h}$ corresponding to the springs standing for the cell–ECM complex are formulated as

2.19a $$\begin{array}{rl} U^{s} & =\frac{1}{2}K_{s}\Delta_{s}^{2}=\frac{1}{2}K_{s}\alpha^{2}\Delta^{2}\\ & =\frac{1}{2}K_{s}\alpha^{2}{\left[L_{t}^{h}(1+\eta -\lambda_{t}^{*}\;{\hat{\lambda }}_{t}^{h}\;f_{2})\right]}^{2} \end{array}$$

and

2.19b $$\begin{array}{rl} U^{h} & =\frac{1}{2}K_{h}\Delta_{h}^{2}=\frac{1}{2}K_{h}{\left(1-\alpha \right)}^{2}\Delta^{2}\\ & =\frac{1}{2}K_{h}{\left(1-\alpha \right)}^{2}{\left[L_{t}^{h}(1+\eta -\lambda_{t}^{*}\;{\hat{\lambda }}_{t}^{h}\;f_{2})\right]}^{2}, \end{array}$$

where, for the effective stiffness of the whole adhesion complex in figure 1*f*, formed by the focal adhesion plaque, the integrins and the substrate, the following expression is adopted [39]:

2.20 $$\begin{array}{rl} K_{\;j} & =\frac{d_{i}(k_{\;p}+k_{\;j})}{L_{c,j}}{\left[\frac{L_{\;p}}{L_{c,j}}+2\text{csch}\frac{L_{\;p}}{L_{c,j}}+\left(\frac{k_{\;p}}{k_{\;j}}+\frac{k_{\;j}}{k_{\;p}}\right)\coth\frac{L_{\;p}}{L_{c,j}}\right]}^{-1}, \end{array}$$

where

2.21 $$L_{c,j}=d_{i}{\left[\frac{k_{\;p}k_{\;j}}{k_{i}(k_{\;p}+k_{\;j})}\right]}^{1/2},$$

with *j* = *h*, *s*. The effective stiffness (2.20) depends on the plaque stiffness *k*_*p*_ = *E*_*p*_*A*_*p*_/*d*_*i*_, on the length of the adhesion plaque *L*_*p*_, the Young modulus *E*_*p*_ and cross-sectional area *A*_*p*_, on the integrins’ average spacing *d*_*i*_ and stiffness *k*_*i*_, and on the stiffness of the substrate *k*_*j*_.

Large angle variations of the rotational spring are allowed so that a nonlinear rotational spring is purposely used, whose elastic energy is cast as [34]

2.22 $$U_{sp}=-2\kappa \ln\left|\cos\frac{\Delta \phi }{2}\right|,$$

where *κ* = *π*^2^ *B*_*t*_/*L* is the rotational stiffness constant, *B*_*t*_ being the bending stiffness of the microtubule groups. By compatibility, the angle Δϕ is written as

2.23 $$\Delta \phi =\phi_{h}+\phi_{s}=\phi_{h}+\arccos\frac{\;{\hat{\lambda }}_{t}^{h}}{\eta \;{\hat{\lambda }}_{t}^{s}}\;f_{1}.$$

The total potential energy of the developed mechanical system gathering all the elastic energy contributions takes the form

2.24 $$P(\alpha ,\;{\hat{\lambda }}_{t}^{h},\;{\hat{\lambda }}_{t}^{s},\phi_{h})=U_{t}^{h}+U_{t}^{s}+2U_{\;f}+U_{sp}+U^{h}+U^{s}.$$

The solution of the problem associated with the vanishing of the first variation of P then provides the stationarity conditions of P with respect to the Lagrangian parameters

2.25 $$\frac{\partial P}{\partial \alpha }=\frac{\partial P}{\partial \phi_{h}}=\frac{\partial P}{\partial \;{\hat{\lambda }}_{t}^{h}}=\frac{\partial P}{\partial \;{\hat{\lambda }}_{t}^{s}}=0.$$

The first stationarity equation readily gives

2.26 $$\alpha =\frac{K_{h}}{K_{s}+K_{h}}.$$

When computing the remaining stationarity conditions, the trivial solution associated with the path sin*ϕ*_*h*_ = 0 is obtained, which corresponds to straight configurations of the system. After discarding the trivial solution and replacing $L_{t}^{s}=\eta L_{t}^{h}$, the second, third and fourth stationarity conditions (2.25) for a generally buckled system state are cast as follows:

2.27a $$\begin{array}{rl} & a_{\eta }\lambda_{\;f}^{*}\;f_{3}\left[\frac{{\left(\;{\hat{\lambda }}_{t}^{h}\lambda_{t}^{*}\right)}^{2}}{{\left(\lambda_{\;f}^{*}\right)}^{2}{\left(1+\eta \right)}^{2}}\;f_{2}-\frac{\lambda_{\;f}^{*}(1+\eta )}{\;{\hat{\lambda }}_{t}^{h}\lambda_{t}^{*}}\frac{1}{{\;f_{2}}^{2}}\right]\\ & \;-A_{k}{\left(L_{t}^{h}\right)}^{2}\;{\hat{\lambda }}_{t}^{h}\lambda_{t}^{*}\;f_{3}\;f_{5}-\kappa \left(\frac{\;{\hat{\lambda }}_{t}^{h}\cos\phi_{h}}{\eta \;{\hat{\lambda }}_{t}^{s}\;f_{1}\;f_{4}}+\frac{1}{\sin\phi_{h}}\right)\tan\frac{\Delta \phi }{2}=0, \end{array}$$

2.27b $$\begin{array}{cc} \; & a_{\eta }\lambda_{\;f}^{*}[-\frac{{\left(\eta {\hat{\lambda }}_{t}^{s}\lambda_{t}^{*}\right)}^{2}}{{\left(\lambda_{\;f}^{*}\right)}^{2}{\hat{\lambda }}_{t}^{h}{\left(1+\eta \right)}^{2}}\frac{\;f_{2}}{\;f_{1}}+\frac{{\hat{\lambda }}_{t}^{h}{\left(\lambda_{t}^{*}\;f_{2}\right)}^{2}}{{\left(\lambda_{\;f}^{*}\right)}^{2}{\left(1+\eta \right)}^{2}}\;\\ \; & \;-\frac{\lambda_{\;f}^{*}(1+\eta )}{{\left({\hat{\lambda }}_{t}^{h}\right)}^{2}\lambda_{t}^{*}\;f_{2}}+\frac{\lambda_{\;f}^{*}(1+\eta ){\left(\eta {\hat{\lambda }}_{t}^{s}\right)}^{2}}{{\left({\hat{\lambda }}_{t}^{h}\right)}^{4}\lambda_{t}^{*}\;f_{1}\;f_{2}^{2}}]+b\lambda_{t}^{*}\left[{\hat{\lambda }}_{t}^{h}-\frac{1}{{\left({\hat{\lambda }}_{t}^{h}\right)}^{2}}\right]\;\\ \; & \;-\kappa \;f_{4}^{-1}\left[\frac{\;f_{1}}{\eta {\hat{\lambda }}_{t}^{s}}-\frac{\eta {\hat{\lambda }}_{t}^{s}}{{\left({\hat{\lambda }}_{t}^{h}\right)}^{2}\;f_{1}}\right]\tan\frac{\Delta \phi }{2}\;\\ \; & \;+A_{k}{\left(L_{t}^{h}\right)}^{2}\lambda_{t}^{*}\left[\frac{{\left(\eta {\hat{\lambda }}_{t}^{s}\right)}^{2}}{{\left({\hat{\lambda }}_{t}^{h}\right)}^{2}\;f_{1}}-\;f_{2}\right]\;f_{5}=0\;\\ \mathrm{and}\; & \frac{a_{\eta }\eta^{2}\lambda_{\;f}^{*}{\hat{\lambda }}_{t}^{s}}{\;f_{1}}\left[\frac{{\left(\lambda_{t}^{*}\right)}^{2}\;f_{2}}{{\left(\lambda_{\;f}^{*}\right)}^{2}{\left(1+\eta \right)}^{2}}-\frac{\lambda_{\;f}^{*}(1+\eta )}{{\left({\hat{\lambda }}_{t}^{h}\right)}^{3}\lambda_{t}^{*}\;f_{2}^{2}}\right]\;\\ \; & +b\eta \lambda_{t}^{*}\left[{\hat{\lambda }}_{t}^{s}-\frac{1}{{\left({\hat{\lambda }}_{t}^{s}\right)}^{2}}\right]-\kappa \;f_{4}^{-1}\left[\frac{\eta }{{\hat{\lambda }}_{t}^{h}\;f_{1}}-\frac{{\hat{\lambda }}_{t}^{h}\;f_{1}}{\eta {\left({\hat{\lambda }}_{t}^{s}\right)}^{2}}\right]\tan\frac{\Delta \phi }{2}\; \end{array}$$

2.27c $$\;-A_{k}{\left(\eta L_{t}^{h}\right)}^{2}\;{\hat{\lambda }}_{t}^{s}\lambda_{t}^{*}\;f_{5}{\left(\;{\hat{\lambda }}_{t}^{h}\;f_{1}\right)}^{-1}=0.$$

In the previous equations, *f*_3_ and *f*_4_ are, in fact, functions of the Lagrangian parameters as follows:

2.28 $$\begin{array}{cc} \;\;\;f_{3}(\phi_{h},\;{\hat{\lambda }}_{t}^{h},\;{\hat{\lambda }}_{t}^{s}) & \;:=\frac{\cos\phi_{h}}{\;f_{1}(\phi_{h},\;{\hat{\lambda }}_{t}^{h},\;{\hat{\lambda }}_{t}^{s})}+1,\\ \;\;\;f_{4}(\phi_{h},\;{\hat{\lambda }}_{t}^{h},\;{\hat{\lambda }}_{t}^{s}) & \;:={\left[1-\frac{{\left(\;{\hat{\lambda }}_{t}^{h}\right)}^{2}}{\eta^{2}{\left(\;{\hat{\lambda }}_{t}^{s}\right)}^{2}}{\;f_{1}(\phi_{h},\;{\hat{\lambda }}_{t}^{h},\;{\hat{\lambda }}_{t}^{s})}^{2}\right]}^{1/2}\;\;\\ \mathrm{and}\;\;f_{5}(\phi_{h},\;{\hat{\lambda }}_{t}^{h},\;{\hat{\lambda }}_{t}^{s}) & \;:=1+\eta -\;{\hat{\lambda }}_{t}^{h}\lambda_{t}^{*}\;f_{2}(\phi_{h},\;{\hat{\lambda }}_{t}^{h},\;{\hat{\lambda }}_{t}^{s}), \end{array}\}$$

though the arguments have been dropped for conciseness. Finally, in equation (2.27), the positions

2.29 $$\begin{array}{r} A_{k}\;:=K_{s}\alpha^{2}+K_{h}{\left(1-\alpha \right)}^{2},\;a_{\eta }\;:=\frac{K_{\;f}L_{t}^{h}(1+\eta )}{3},\;b\;:=\frac{K_{t}L_{t}^{h}}{3} \end{array}$$

have been set and the relationship (2.23) has been exploited.

The system of three nonlinear equations in the three unknowns $\phi_{h},\;\;{\hat{\lambda }}_{t}^{h},\;\;{\hat{\lambda }}_{t}^{s}$ has been numerically solved by using the nonlinear systems solver *fsolve* available in Matlab^©^. Then, the angle \phi_s and the displacement Δ have been computed via equations (2.7) and (2.14), while $\Delta_{s}$ and $\Delta_{h}$ have been obtained through equations (2.16) and (2.26).

### 2.4. Force on the focal adhesion plaque

Durotaxis manifests as a consequence of the fact that the cell contractility generates forces on the substrate, and these forces must be balanced either at the cell periphery or throughout the cell–substrate interface [18].

In the present model, the mechanical force exerted from the cell on the focal adhesion plaque is obtained as

2.30 $$F_{\;p}=K_{s}\Delta_{s}=K_{h}\Delta_{h}.$$

It can be drawn from equation (2.30) that the plaque force *F*_*p*_ is proportional to both the stiffness of the focal adhesions–substrate complex (2.20), which also depends on the plaque length *L*_*p*_, and the values of $\Delta_{s}$ and $\Delta_{h}$ computed through equation (2.16), the latter being influenced by the asymmetry ratio *η*. The specific form taken by *F*_*p*_ and its relevance to the plaque’s attitude towards assembly and disassembly will be thoroughly addressed in §7.2.

### 2.5. Growth rate of the focal adhesion plaque

For the derivation of the law governing the growth rate of the focal adhesions (FAs), we refer to the previous authors’ contribution [33], where, analogously to the tensegrity components, the plaque is assumed to be made of a material obeying a neo-Hookean law. Let *λ*_*p*_ be its stretch, and ${\tilde{\lambda }}_{\;p}$ its approximated expression through a second-degree Taylor polynomial in terms of the force *F*_*p*_ exerted by the system on the plaque around *F*_*p*_ = 0,

2.31 $${\tilde{\lambda }}_{\;p}=\frac{F_{\;p}^{2}}{k_{\;p}^{2}h_{\;p}^{2}}+\frac{F_{\;p}}{k_{\;p}h_{\;p}}+1,$$

where *k*_*p*_ and *h*_*p*_ denote the plaque stiffness and height, respectively. We assume a chemical potential *μ*_*p*_ for the plaque and consider that, by virtue of the thermodynamical laws, an infinitesimal variation of the force acting on the plaque, *F*_*p*_, induces an infinitesimal variation *dμ*_*p*_ as follows [42,43]:

2.32 $$d\mu_{\;p}=-l_{m}(F_{\;p})dF_{\;p}=-{\tilde{\lambda }}_{\;p}(F_{\;p})d_{i}dF_{\;p},$$

where $l_{m}(F)={\tilde{\lambda }}_{\;p}(F_{\;p})d_{i}$ is the actual length of the single molecular constituent of the plaque and *d*_*i*_ is the molecular length of each monomer at rest.

Let $\mu_{\;p}^{0}$ be the chemical potential characterizing the plaque subunit without mechanical forces. The chemical potential at the reference position coordinate *X* is obtained by integration of equation (2.32) as

2.33 $$\mu_{\;p}(X)=\mu_{\;p}^{0}-d_{i}\int_{0}^{F_{\;p}(X)}{\tilde{\lambda }}_{\;p}(f_{\;p})\;df_{\;p}.$$

The difference Δμ between the chemical potentials *μ*_*p*_(*X*) and *μ*_free_ associated with the bounded and the free molecules is cast as [33]

2.34 $$\Delta \mu (X)=\Delta \mu_{0}-d_{i}\int_{0}^{F_{\;p}(X)}{\tilde{\lambda }}_{\;p}(f_{\;p})\;df_{\;p},$$

where $\Delta \mu_{0}=\mu_{\;p}^{0}-\mu_{\mathrm{free}}$ is the chemical potential variation at vanishing force. Δμ drives the transfer of monomers between the plaque and the surroundings, as non-assembled molecules will join or abandon the plaques with negative and positive Δμ, respectively.

The plaque is taken free at the left end, i.e. *F*_*p*_ (0) = 0, while the right side is loaded by the axial force exerted by the mechanical system. Assuming the local molecular flux towards the plaque of the form

2.35 j(X)=−DΔμ(X),

with *D* a positive coefficient governing the assembly kinetics, the total growth rate of the adhesion plaque can be obtained as

2.36 $$J=-D\left[2\Delta \mu_{0}-d_{i}\int_{0}^{F_{\;p}(L_{\;p})}{\tilde{\lambda }}_{\;p}(f_{\;p})\;df_{\;p}\right],$$

assuming molecular exchanges occur at the sole plaque ends. Noteworthy, positive and negative values of the flux *J* will correspond to the assembly and disassembly of the monomers, respectively.

## 3. Parameters and critical pre-strains

The results shown in the forthcoming sections have been obtained by adopting the parameters in table 1 and aim to show how the substrate stiffness gradients and geometrical asymmetry affect the cell behaviour.

**Table 1.** Adopted geometrical and constitutive parameters of the cell-equivalent model and corresponding realistic ranges. Herein MT and SF identify the microtubule and the stress fibre, respectively.

parameter	description	value	source	typical value
*L*	MT and SF rest length	20 μm	[32]	10–50 μm
*L*_*p*_	adhesion plaque rest length	variable	[39]	up to few μm
*h*_*p*_	plaque height	100 nm	[44]	50–100 nm
*w*_*n*_	plaque width	1000 nm	[44]	1000 nm
*d*_*i*_	integrin spacing	100 nm	[45]	100 nm
*A*_*t*_	MT rest cross-sectional area	190 nm^2^	[46,47]	190 nm^2^
*A*_*f*_	SF rest cross-sectional area	10^4^*π* nm^2^	[46]	10^4^*π* nm^2^
*E*_*t*_	MT Young modulus	1.2 GPa	[32]	1.2 GPa
*E*_*f*_	SF Young modulus	1.45 MPa	[46]	1.45 MPa
*k*_*i*_	integrin stiffness	5 pN nm^−1^	[48]	5 pN nm^−1^
*k*_*p*_	plaque stiffness	2.5 pN nm^−1^	[49,50]	2.5 pN nm^−1^
$\Delta \mu_{0}$	energy barrier for protein recruitment without mechanical load	250 *k*_B_*T*	[51]	10–250 *k*_B_*T*
*B*_*t*_	MT bending stiffness	15 nN μm^2^	[52]	0.0215–215 nN μm^2^

In the applications hereafter reported, the parameter *χ* = *k*_*s*_/*k*_*h*_ indicates the ratio between soft and hard substrate moduli, while the value of the average stiffness *k*_av_ between the values of *k*_*s*_ and *k*_*h*_ is assumed to vary so that the smaller the *χ*-value the larger the stiffness gradient. Hence the stiffness values are determined once the values of *χ* and *k*_av_ are known.

### 3.1. Choice of the pre-strain values

**Figure 2.**
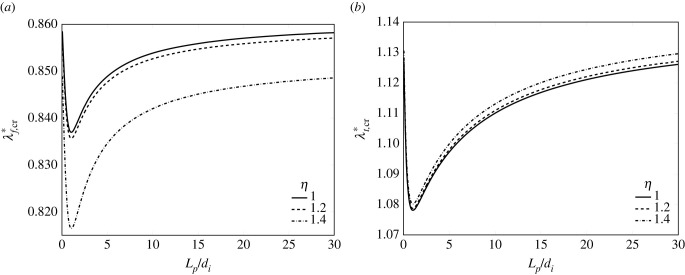
Profiles of $\lambda_{\;f,\mathrm{cr}}^{*}$ (*a*) and $\lambda_{t,\mathrm{cr}}^{*}$ (*b*) as a function of the normalized plaque length *L*_*p*_/*d*_*i*_. The curves refer to three different values of asymmetry, setting *χ* = 0.01, *k*_av_ = 100 pN nm^−1^.

For the subsequent developments, it is crucial to identify the critical values of the pre-strains, hereafter referred to as $\lambda_{\;f,\mathrm{cr}}^{*}$ and $\lambda_{t,\mathrm{cr}}^{*}$, that is, the pre-strains at which the equilibrium paths of the tensegrity system bifurcate, thus allowing for a possible switch from straight to buckled configurations. These critical values have been purposely computed by solving the stationarity conditions for variable pre-strain values. Figure 2*a*,*b* reports the trends of $\lambda_{\;f,\mathrm{cr}}^{*}$ and $\lambda_{t,\mathrm{cr}}^{*}$, respectively, for variable normalized plaque length *L*_*p*_/*d*_*i*_. For a correct interpretation of the figure, it should be kept in mind that the buckled configuration is reached when the actomyosin contraction assumes values smaller than $\lambda_{\;f,\mathrm{cr}}^{*}$ or the polymerization stretch is larger than $\lambda_{t,\mathrm{cr}}^{*}$, when the two pre-strains are independently applied as considered in figure 2. The shown profiles correspond to symmetric configuration, *η* = 1, and two asymmetric configurations, that is for *η* = 1.2, 1.4. On an observational basis, the critical stretches are nonlinear functions of *L*_*p*_. However, the lack of symmetry slightly decreases $\lambda_{\;f,\mathrm{cr}}^{*}$ and increases $\lambda_{t,\mathrm{cr}}^{*}$. Thus, an asymmetric system requires higher pre-contraction or pre-polymerization levels to achieve buckling compared with a symmetric one.

Based on the previous outcomes, the pre-strain values to be used to trigger buckling in the mechanical system have been properly selected. In particular, $\lambda_{f}^{*}=0.882$ and $\lambda_{t}^{*}=1.1$ have been adopted in the applications shown in the forthcoming sections, as they represent values able to trigger buckling, that is, pericritical values of pre-contraction and pre-polymerization, respectively.

**Figure 3.**
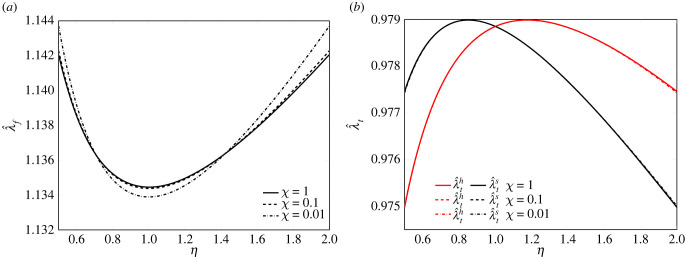
Incremental stretch of the actomyosin bundle (*a*) and incremental stretches of the microtubule subunits $\lambda_{t}^{s}$ and $\lambda_{t}^{h}$ (*b*) plotted as a function of the asymmetry ratio *η*. The curves refer to three different values of ratio *χ*, setting *L*_*p*_ = 2*d*_*i*_, *k*_av_ = 100 pN nm^−1^, with underlying pre-contraction $\lambda_{\;f}^{*}=0.882$ and pre-polymerization $\lambda_{t}^{*}=1.1$.

To the prescribed pre-stretches, the mechanical system responds with incremental stretches ${\hat{\lambda }}_{\;f}$, ${\hat{\lambda }}_{t}^{h}$ and ${\hat{\lambda }}_{s}^{h}$, shown in figure 3*a*,*b*. Particularly, the actomyosin fibre bundle ${\hat{\lambda }}_{\;f}$ is sensitive to both the substrate stiffness gradient and the lack of symmetry of the system, as figure 3*a* proves. On the contrary, figure 3*b* highlights that the microtubule elastic stretches are affected by the non-symmetry of the system while being almost independent of the substrate stiffness. This can be observed by comparing the profiles of the elastic incremental stretches of the microtubule subunits at the soft-substrate side ${\hat{\lambda }}_{t}^{s}$, in black, and at the hard-substrate side ${\hat{\lambda }}_{t}^{h}$, in red, for variable *η*.

## 4. Asymmetry can turn positive durotaxis into negative durotaxis for certain substrate stiffness gradients

The present section aims to assess the dependence of the kinematics of the cell system on both geometrical asymmetry and substrate stiffness gradients for a fixed length of the adhesion plaque *L*_*p*_.

By virtue of geometric compatibility and by considering the eccentric position of the rotational hinge, the buckling angles *ϕ*_*s*_ and *ϕ*_*h*_ at the cell edges turn out to be generally different, the symmetric case being recovered for *η* = 1, when *ϕ*_*s*_ = *ϕ*_*h*_. However, it has been verified that, for the present combination of inelastic pre-stretches, these buckling angles are almost unaffected by the stiffness gradient of the substrate.

On the other hand, both the displacements $\Delta_{s}$ and $\Delta_{h}$ at the rear and front of the system and the net displacement $\Delta_{N}$ are strongly influenced by both asymmetry and substrate stiffness. In particular, these displacements have been computed both for variable asymmetry ratio *η* ∈ [0.5, 2] and different stiffness of the substrate, while setting the average stiffness of the substrate to the value *k*_av_ = 100 pN nm^−1^. Furthermore, pre-contraction of the fibres $\lambda_{f}^{*}=0.882$ and pre-polymerization $\lambda_{t}^{*}=1.1$ have been prescribed.

**Figure 4.**
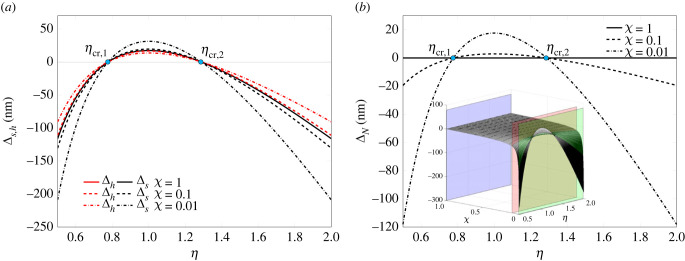
Displacements of the system at cell edges (*a*) and net displacement of the cell (*b*) plotted as a function of the asymmetry ratio *η*. Here, $\Delta_{N}>0$ indicates positive durotaxis, while $\Delta_{N}<0$ indicates negative durotaxis. The curves refer to three different values of ratio *χ*, setting *L*_*p*_ = 2*d*_*i*_, *k*_av_ = 100 pN nm^−1^, with underlying pre-contraction $\lambda_{\;f}^{*}=0.882$ and pre-polymerization $\lambda_{t}^{*}=1.1$.

In this regard, figure 4*a* shows the profiles of the displacements of the system at the soft substrate side, $\Delta_{s}$, in black, and at the hard substrate side, $\Delta_{h}$, in red, in terms of the asymmetry ratio *η*. Three different values of the ratio *χ* = *k*_*s*_/*k*_*h*_ have been used, so that the smaller *χ* the higher the stiffness gradient. Remarkably, for a fixed value of the average substrate stiffness, the maximum displacements of the system occur at *η* = 1.

The system durotactic attitude can be drawn from the profiles of the net displacement $\Delta_{N}$ in figure 4*b*. It can be observed that $\Delta_{N}$ vanishes at two distinct critical values of *η*, i.e. *η*_cr,1_ and *η*_cr,2_, which also make vanishing both the values of $\Delta_{s}$ and $\Delta_{h}$, as shown in figure 4*a*. These critical values of *η* are referred to as critical asymmetry ratios, because they imply a transition from positive to negative of the sign of the net displacement. Particularly, positive values of $\Delta_{N}$ for *η* belonging to the interval [*η*_cr,1_, *η*_cr,2_] in figure 4*b*, elicit positive durotaxis, that is, displacements towards the stiff side, while negative values of $\Delta_{N}$ indicate that the system moves towards the soft side, a circumstance that leads to negative durotaxis and that occurs for values of *η* outside the aforementioned interval of asymmetry ratios. For instance, for $\lambda_{f}^{*}=0.882$ and $\lambda_{t}^{*}=1.1$, the two critical ratios are equal to 0.775 and 1.289. To further highlight the dependence of the durotaxis on both the present asymmetry sources, namely geometry and substrate gradient, a three-dimensional view of the system displacement $\Delta_{N}$ in terms of the asymmetry ratios *η* and the substrate stiffness ratio *χ* can be appreciated in the inset figure of figure 4*b*. Particularly, the surface of $\Delta_{N}$ is cut by three planes: the green plane, at *χ* = 0.01 for a large stiffness gradient, the red plane, for *χ* = 0.1 when the maximum displacement for positive durotaxis decreases, and the violet plane, for *χ* = 1, when the profile of $\Delta_{N}$ is quite flat and durotaxis is neutral as the edge displacements are equal and opposite. The profile of the intersection of $\Delta_{N}$ with the planes for variable *χ* coincides in fact with that previously shown in figure 4*b*. For fixed pre-contraction and pre-polymerization levels, however, the critical *η* values remain constant, hence, the durotaxis domain in terms of *η* remains unaltered. The $\Delta_{N}$ profiles share the same critical values *η*_cr,1_ and *η*_cr,2_ for variable *χ*. Thus, the question arises of what is the main factor governing these critical values. Preliminary tests indeed suggest investigating how the values of the critical values *η*_cr,1_ and *η*_cr,2_ change for variable pre-strains. This issue is commented on in §5.

## 5. Critical asymmetry ratios depend on pre-strains

To systematically study the dependence of the critical asymmetry ratios *η*_cr,1_ and *η*_cr,2_ on the pre-strains, the equilibrium problem has been reformulated to take into account that the critical condition at which the system switches from positive to negative durotaxis also corresponds to $\Delta =\Delta_{s}+\Delta_{h}=0$, Δ being given by equation (2.14). Hence, the solution of the stationarity of $P(\;{\hat{\lambda }}_{t}^{h},\;{\hat{\lambda }}_{t}^{s},\phi_{h})$ has been solved under the constraint Δ=0, so that the latter condition replaces the first stationary condition ∂P/∂α=0 in equation (2.25) and the parameter *η* turns out being unknown.

**Figure 5.**
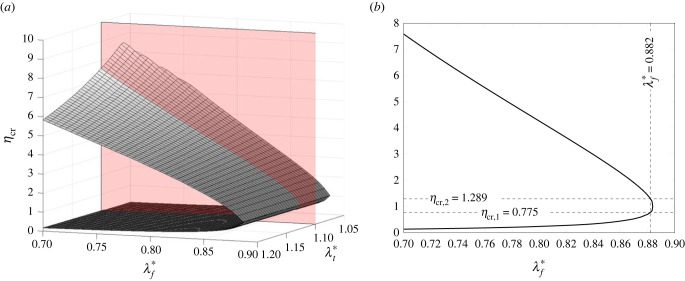
Critical values of *η* as a function of both $\lambda_{t}^{*}$ and $\lambda_{f}^{*}$ (*a*) and at fixed $\lambda_{t}^{*}=1.1$ (*b*) with *L*_*p*_ = 2*d*_*i*_, *k*_av_ = 100 pN nm^−1^.

The numerical solution of the system of the aforementioned nonlinear equations in the four unknowns $\left(\eta ,\phi_{h},\;\;{\hat{\lambda }}_{t}^{h},\;\;{\hat{\lambda }}_{t}^{s}\right)$ is shown in figure 5*a* in terms of *η*, i.e. *η*_cr_ , versus the pre-strain values. The solution obtained at $\lambda_{t}^{*}=1.1$ for variable *λ*_f_*, belonging to the plane in red in figure 5*a*, is then plotted in figure 5*b*. This latter figure highlights the two critical values that define the relevant interval of positive durotaxis. The profile is not significantly affected by the value of $\lambda_{t}^{*}$.

The aforementioned values *η*_cr,1_ = 0.775 and *η*_cr,2_ = 1.289 are expectedly found at *λ*_f_* = 0.882. However, it can be further observed that while *η*_cr,2_ increases for higher pre-contraction levels, *η*_cr,1_ reduces. Hence, the interval of positive durotaxis enlarges for increasing pre-contraction levels, while negative durotaxis requires higher or lower values of *η* to manifest. On the other hand, for values larger than 0.89, buckling does not occur and durotaxis is not triggered. However, when a small pre-contraction level is assumed, the system is on the verge of negative durotaxis for small perturbations of the system geometrical symmetry. Pre-contraction and pre-polymerization, indeed, produce cross-current tendencies as the system will exert pushing or pulling forces on the plaque depending also on the balance between these competing pre-strains. Hence, in case of combinations of the pre-strains with prevailing pre-polymerization, negative durotaxis may emerge, otherwise, positive durotaxis will manifest.

## 6. Asymmetry and stiffness gradient influence plaque force and growth

We investigate the response of the plaque to a simultaneous lack of symmetry and stiffness gradient presence in terms of the forces exerted on the focal adhesion complexes and the related plaque growth flux.

**Figure 6.**
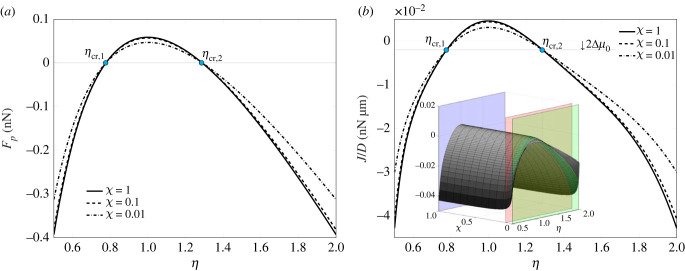
Force *F*_*p*_ (*a*) and growth law J of the adhesion plaque (*b*) plotted as a function of the asymmetry ratio *η*. The curves refer to three different values of ratio *χ* and *k*_av_ = 100 pN nm^−1^, setting *L*_*p*_ = 2*d*_*i*_, with underlying pre-contraction $\lambda_{f}^{*}=0.882$and pre-polymerization $\lambda_{t}^{*}=1.1$.

Figure 6*a*,*b* displays the force on the plaque, *F*_*p*_, and the net plaque growth flux, *J* defined by equation (2.36), respectively, as functions of the asymmetry ratio *η*. The three curves for different values of the stiffness ratio *χ* have been obtained for underlying pre-contraction and pre-polymerization *λ*_f_* = 0.882 and $\lambda_{t}^{*}=1.1$, respectively. In particular, the three-dimensional surface of *J*/*D* as a function of both *η* and *χ* is shown as an inset in figure 6*b*. Its intersection with the planes corresponding to *χ* = 1, 0.1, 0.01 coincides with the two-dimensional profiles in figure 6*b*.

Remarkably, the case *η* = 1 corresponding to a symmetric configuration is associated with positive fluxes. Thus, a symmetric system will imply a non-equilibrium state of the plaque. Therefore, it can be observed that the system may switch from a symmetric configuration to an asymmetric one to reach equilibrium. The same concept could be rephrased by saying that asymmetric buckling is one of the options which the system can leverage to influence the focal adhesion plaque stability.

Because of the shift of *J* with respect to its aliquot depending on the force, induced by the constant term $2\Delta \mu_{0}$ (see equation (2.36)), positive (negative) durotaxis and force pulling (pushing) on the plaque do not strictly imply plaque assembly (disassembly). As a consequence, the critical *η*-values at which $\Delta_{N}$ and *J* vanish do not coincide. However, all the profiles of *J* intersect the horizontal axis shifted down of $2\Delta \mu_{0}$ at points *η*_cr,1_ and *η*_cr,2_ for any stiffness ratio *χ*.

### 6.1. Remarks

Figures 4*b* and 6*b* highlight three main stages of the durotaxis and the net growth flux for variable asymmetry ratio. In particular, the curves of $\Delta_{N}$ and *F*_*p*_ intercept the abscissa at two critical *η*, *η*_cr,1_ and *η*_cr,2_, whose values turn out being independent of the stiffness gradient *χ*.

As previously mentioned, the same remarks do not strictly apply to *J*, since it vanishes at values of *η* which are different from the aforementioned *η*_cr,1_ and *η*_cr,2_ discriminating positive from negative durotaxis. Nevertheless, the critical *η* values of J are key to the plaque stability, as they correspond to equilibrium states of the plaque where neither assembly nor disassembly takes place, any other *η* corresponding instead to either positive or negative fluxes. On assuming that the system can leverage both *L*_*p*_ and *η* to reach equilibrium states of the plaques, the observed findings can be clarified as follows.

*η* < *η*_cr,1_. The flux *J* is negative. The plaque is not in equilibrium, and will expectedly disassemble up to disappear or reach a new stable length, at which neither assembly nor disassembly takes place. Alternatively, the system can leverage asymmetric buckling and increase the asymmetry ratio *η* to approach the closest equilibrium position.
*η*_cr,1_ ≤ *η* ≤ *η*_cr,2_. The flux *J* is part positive and part negative. The plaque will accordingly tend to a new length at which neither assembly nor disassembly is allowed. Alternatively, the plaque may reach an equilibrium state by moving the position of the hinge.
*η* > *η*_cr,2_. The flux *J* is negative, thus the plaque is not in equilibrium. The plaque will either depolymerize up to a stable length or disappear; alternatively, the system can decrease *η* to tend to the closest equilibrium state.

## 7. The role of plaque geometry and average substrate stiffness

When addressing durotaxis, state-of-the-art experimental studies often report parameters related to the mechanical behaviour of adherent cells detected at variable average substrate stiffness [21]. Therefore, to gain a better insight into the role played by the average mechanical stiffness of the substrate in determining the model response, the sensitivity of the mechanical response to the plaque length *L*_*p*_ and the parameter *k*_av_ is investigated in §§7.1 and 7.2, respectively. In the case of symmetric buckling and stiff homogeneous substrates, the system reportedly requires larger stable plaque lengths with respect to the case of soft substrates [33]. In §7.1, the concurrent effect of asymmetrical buckling and stiffness gradient is considered by assuming constant *k*_av_ and variable *L*_*p*_. In §7.2, besides the influence of *k*_av_ for variable *χ* and *η* is studied, because the average substrate information is not *per se* sufficient and must be complemented by the knowledge of the stiffness ratio to fully characterize the substrate stiffness.

### 7.1. The influence of the plaque length

**Figure 7.**
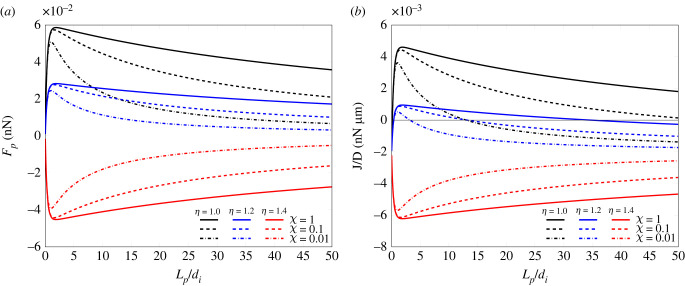
Force *F*_*p*_ (*a*) and growth law *J* of the adhesion plaque (*b*) plotted as a function of the plaque length *L*_*p*_/*d*_*i*_ with *k*_av_ = 100 pN nm^−1^ and underlying pre-contraction $\lambda_{f}^{*}=0.882$ and pre-polymerization $\lambda_{t}^{*}=1.1$.

Besides the symmetric reference solution for the symmetric case at *η* = 1, two other values of *η* were taken, *η* = 1.2, 1.4, which are pre-critical and post-critical values according to the evaluations performed. The results show that both asymmetry and stiffness gradient strongly affect the values taken by both *F*_*p*_, shown in figure 7*a*, and the flux *J*, plotted in figure 7*b*.

Both the figures have been plotted at fixed *k*_av_ = 100 pN nm^−1^ and assuming the stiffness ratio *χ* = 1, 0.1, 0.01, where *χ* = 1 corresponds to the uniform substrate, and smaller values of *χ* refer to higher substrate stiffness gradients. On an observational basis, from figure 7*b*, it can be inferred that the stable plaque length decreases for increasing asymmetry on a uniform substrate, *χ* = 1. Analogously, in symmetric or slightly asymmetrical systems, with *η* = 1 and *η* = 1.2, respectively, the stable lengths decrease for enhancing stiffness gradients, namely, decreasing *χ*. In the case of markedly asymmetrical systems, corresponding to the profile with *η* = 1.4 in the figure, the growth flux is always negative and the plaque can never reach equilibrium, and this independently of the stiffness ratio *χ* characterizing the substrate.

### 7.2. Plaque force and growth depend on the average stiffness

**Figure 8.**
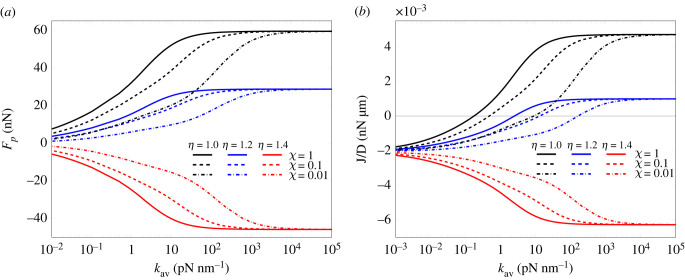
Force *F_p_* (*a*) and growth law *J* of the adhesion plaque (*b*) plotted as a function of the substrate average stiffness *k*_av_ in a semi-logarithmic plot. The curves have been obtained setting *L*_*p*_ = 2*d*_*i*_, with underlying pre-contraction $\lambda_{f}^{*}=0.882$, and pre-polymerization $\lambda_{t}^{*}=1.1$.

For the purpose of assessing the role played by both the average substrate stiffness and stiffness gradient on the plaque force and growth law, these have been plotted in a semi-logarithmic scale in figure 8*a*,*b*, respectively, for the same *χ* and *η* values adopted in the previous section and increasing substrate average stiffness *k*_av_. The magnitude of the force exerted by the system on the adhesion plaque is an increasing function of the substrate average stiffness *k*_av_ and plateaus beyond a critical average stiffness. An analogous trend is exhibited by *J*, the only difference being dictated by the shift of the zero-crossing points due to the threshold $2\Delta \mu_{0}$.

The obtained trend of *F*_*p*_ versus *k*_av_ is in line with literature experimental and analytical results [53]; however, they differ from the biphasic force–stiffness profile exhibited by neurons in the physiological stiffness range, where an ascending path culminates in a peak for an optimal value of the substrate stiffness and is subsequently followed by a descendent path. In other cells, such as glioma cells, this biphasic force–substrate–stiffness behaviour is instead obtained after talin depletion [21].

The dependency of the force on the plaque *F*_*p*_ on the average stiffness must also be discussed in light of the values taken by *η* and *χ*. Based on the obtained profiles, it can be inferred that *F*_*p*_ cannot overcome its asymptotic value. Moreover, symmetric systems on homogeneous gradients exert the maximum absolute value of the force *F*_*p*_ on the plaque.

Moreover, the trends shown by figure 8*a* confirm what is already highlighted in figure 7*a*.

It also shows that pushing forces decrease their value for increasing stiffness ratio so that plaques placed on high soft-to-hard substrate gradients are pushed to a lesser extent than their homologous located on homogeneous environments. On the other hand, pulling forces exerted on the plaque during positive durotaxis are smaller in asymmetric systems, that is, for *η* larger than 1.

With regard to the plaque flux, due to the constant term $-2\Delta \mu_{0}$ in the expression of *J*, there is no direct correspondence between pulling and pushing forces and the plaque assembly and disassembly. Nevertheless, pushing forces always come along with plaque disassembly, and, analogously to what is detected for the force *F*_*p*_, the disassembly flux is weaker on high stiffness gradients than on homogeneous substrates. An opposite tendency emerges in the case of positive flux, where the assembly process intensifies on homogeneous substrates up to a certain value of *k*_av_, beyond which *J* asymptotically tends to a constant value independently from *χ*, and attenuates in asymmetric systems.

The present comparative analysis suggests that asymmetric buckling might be regarded as a weakening of the plaque assembly, while the same remark does not strictly apply to the disassembly process. Furthermore, the plaque disassembly tendency is lower in the presence of strong stiffness gradients as well as of soft average substrate stiffness. With respect to asymmetric buckling, symmetric buckled configurations on homogeneous substrates display the highest assembly and disassembly forces for increasing average stiffness.

## 8. Discussion

In a previous contribution [33], it has been shown that the current model predicts longer focal adhesion plaques when anchored to stiffer substrates than on softer substrates at the same levels of pre-contraction and pre-polymerization. Moreover, it was found that comparatively soft substrates allow for greater cell displacements, with the consequence that this might favour cell mobility.

The present study further suggests that, in the positive durotaxis regime, asymmetric systems solicit smaller stable lengths of the adhesion plaque than symmetric ones.

Though a non-monotonic relationship between focal adhesion size and cell speed was experimentally detected, suggesting that cell motility might both increase or decrease with the plaque length based on the considered cell length range [54], the argument that focal adhesion size, rather than shape, is highly predictive of cell migration speed seems solid [54,55]. Thus, as the plaque length is correlated to the cell mobility, the present results suggest that asymmetry influences locomotion, more markedly when cells lie on hard substrates, at least when positive durotaxis manifests.

Moreover, the higher the stiffness gradient of the substrate, the shorter the focal adhesion complex. Thus, higher substrate gradients also affect cell mobility.

In the present model, the lack of symmetry of the mechanical system generally delays buckling, which, in turn, requires higher pre-contraction or pre-polymerization than those necessary to activate buckling in symmetric systems.

The force acting on the focal adhesion plaque is tensile or compressive for positive and negative displacements, respectively. This aspect holds only for the flux *J* net of the constant $2\Delta \mu_{0}$ contribution. Noteworthy, the lack of symmetry of the system and the stiffness gradient of the substrate strongly affect the overall behaviour of the tensegrity model. The symmetric system is associated with the largest values of the anchorage force, hence confirming the hypothesis that asymmetric systems are more mobile than symmetric ones.

The model suggests that, in general, both the substrate stiffness and its gradient are key to focal adhesion plaques’ stability. In particular, focal adhesion plaques developing on soft substrates possibly undergo smaller growth fluxes than those located on both hard and uniform substrates. This remark adds further insight into the durotaxis mechanism as well as offers inspiration for experimental validation.

## 9. Conclusion

Cell locomotion is an inherently asymmetric, polarized, process. It is also influenced by substrate stiffness gradients.

In the present contribution, two sources of asymmetry were introduced, that is an internal geometrical source through asymmetric buckling of the cell microtubule-like component and an external source accounting for gradients of the substrate stiffness at the cell ends. Particularly, the cell–focal adhesion–substrate complex has been simulated using a nonlinear tensegrity model capable of asymmetric buckling and connected to springs with graded stiffness.

In a nutshell, the system durotaxis, whether positive or negative, turns out to be influenced by the plaque geometry, the substrate stiffness ratio and average stiffness, and, finally, by the asymmetric contractility latent in the tensegrity geometry.

Remarkably, for certain stiffness gradients and suitable combinations of the contraction and polymerization pre-strains, the system can leverage the change of configuration associated with a change of *η* to stabilize focal adhesion plaques, while exhibiting both durotaxis and *mollitaxis*. Particularly, when negative durotaxis takes place, the magnitude of the pushing force on the plaque decreases with decreasing average stiffness. Associating compressive forces with depolymerization [43], comparatively longer plaques would develop on softer substrates. As the focal adhesion plaque length is indicative of the cell spreading on the substrate, cells would, therefore, tend to be more spread on soft substrates in the case of mollitaxis, contrarily to what happens in positive durotaxis. Furthermore, as plaque lengths are related to cell motility, both the lack of symmetry and substrate stiffness gradients may influence cell locomotion.

An ongoing extension of the present model accounts for the elastic-brittle behaviour of the springs representing the focal adhesion–ECM complexes so that, once a critical displacement is reached, the substrate anchorage is released.

## Data Availability

The data underlying the study have been drawn from the literature and are indicated in table 1. The paper uses data published elsewhere. All data have been drawn from the cited literature and are freely available.
